# C. Diff-erently: evaluating the association of inappropriate antibiotic use with hospital-onset *Clostridioides difficile* infection

**DOI:** 10.1017/ash.2025.10161

**Published:** 2025-10-08

**Authors:** Amie John, Antoinette Acbo, Kelsie Cowman, Yi Guo, Priya Nori, Gregory Weston

**Affiliations:** 1 Division of Infectious Diseases, Albert Einstein College of Medicine, Montefiore Medical Center, Bronx, NY, USA; 2 Department of Pharmacy, Albert Einstein College of Medicine, Montefiore Medical Center, Bronx, NY, USA; 3 Network Performance Group, Montefiore Medical Center, Albert Einstein College of Medicine, Bronx, NY, USA

## Abstract

Hospital-onset *C. difficile* infection (HO-CDI) remains a common cause of healthcare-associated infection. This study evaluated antibiotic use (AU) and appropriateness in hospital-onset *C. difficile* cases compared to matched controls in an inpatient setting at a New York City hospital. Elevated or inappropriate AU was not associated with HO-CDI.

## Introduction

Hospital-onset *Clostridioides difficile* infection (HO-CDI) is a common healthcare-associated infection and is associated with increased costs, morbidity, and mortality.^1–3^ Although any antibiotic exposure might increase patient risk for HO-CDI, unnecessary antibiotic use (AU) represents the modifiable portion of that risk. Studies have shown substantial inappropriate AU in patients with CDI, but without comparison to control patients.^4^ This study evaluated the association of inappropriate AU with HO-CDI.

## Methods

This observational, retrospective, case–control study included admitted patients ≥18 years with a CDI test between 1/1/23 and 2/28/24. Testing consisted of initial glutamate dehydrogenase/toxin testing (Techlab, Inc.) with additional PCR (Cepheid GeneXpert) for discordant glutamate dehydrogenase and toxin results. HO-CDI cases were defined by National Healthcare Safety Network criteria.^5^ Controls had a negative CDI test sent on or after hospital day 4 and no positive CDI test within the index admission. In controls with multiple negative CDI tests, the last one was used for analysis. Cases and controls were matched 1:2, when possible, based on patient location (critical care, oncology, or medical-surgical) and month of index test. Inappropriate AU and antibiotic indication were determined by chart review by an infectious diseases (IDs) trained physician or pharmacist. Inappropriate AU was categorized as antibiotic not needed, prolonged antibiotic duration, or inappropriate antibiotic spectrum (Supplement Part A). Appropriateness of AU for infectious syndromes was determined using institutional guidelines (Supplement Parts B, C).^6^ Questionable instances of inappropriate AU or antibiotic indication were adjudicated by additional investigators. Patients could be assigned multiple categories of inappropriateness. The AU rate of all antibiotics and selected high-risk antibiotics^7^—cefepime, ciprofloxacin, levofloxacin, ceftriaxone, and piperacillin-tazobactam—during 30 days prior to the index test was calculated as days of therapy (DOT) per 1000 patient days. Demographics, race and ethnicity, proton pump inhibitor use, prior CDI history, gastrointestinal surgery, hospital length of stay (LOS), and all-cause mortality within 30 days of index test were collected from the electronic health record.

### Sample size calculation

We included eligible cases during the study period that had at least one matching control patient. We used a previous study to estimate inappropriate AU in cases^4^ and calculated that matching cases to controls 1:2 would provide 80% power with *α* = 0.05 to detect an effect size of 45% inappropriate AU in the cases and 28% in controls.

### Statistical analysis

Baseline characteristics and secondary outcomes were summarized using descriptive statistics. Bivariate analyses were conducted via χ2 test, Fisher’s exact test, and Wilcoxon rank sum as appropriate. All tests were two-tailed, and *p–*values < .05 were considered significant. Statistical analysis was conducted using SAS version 9.4 software (SAS Institute, Cary, NC).

## Results

**Figure 1.**
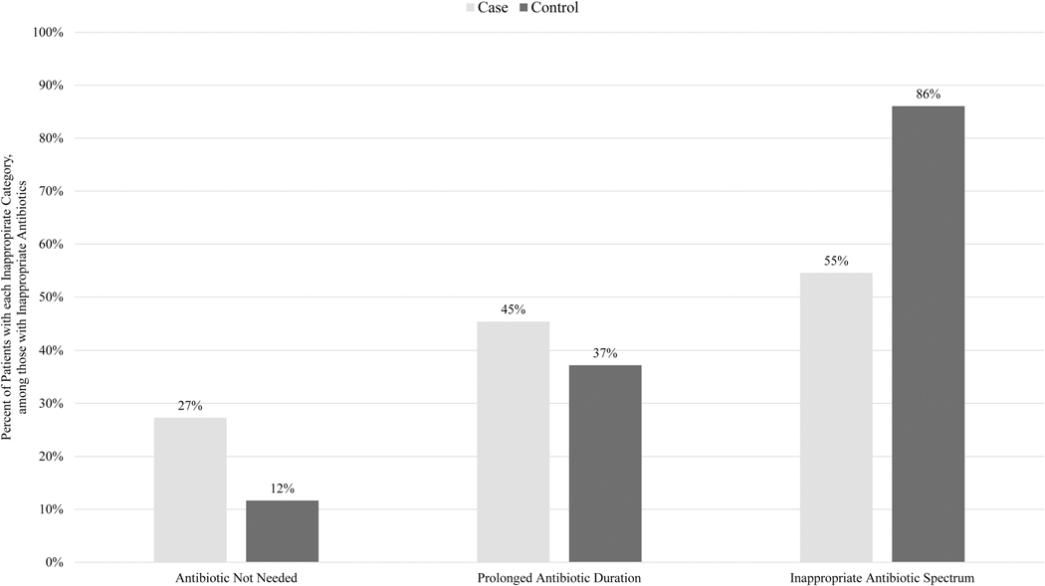
Categories of inappropriate antibiotic use. Denominator of percentage is the number of patients who had inappropriate antibiotic use (case *n* = 22; control *n* = 43). A given patient could have more than one category of inappropriate antibiotic use, and more detailed definitions of each category of inappropriate antibiotic use are available in the supplementary appendix.

Two hundred ninety-six patients were included: 102 cases and 194 controls. Only one matching control could be identified for ten cases. Median age was similar between cases and controls (61 vs 63 yr). Substantial proportions of the population identified as Black (32% cases and 32% controls) or Hispanic (44% cases and 41% controls). There were no significant differences in race or ethnicity distribution between cases and controls. Most patients were admitted to medical-surgical wards (75% cases and 75% controls). The ID service recommended antibiotics in 54% of cases and 46% of controls. AU rate for high-risk antibiotics was lower in cases compared to controls (*P* = .01) (Table 1). However, there was no significant difference between the groups in AU rate for all antibiotics. Inappropriate AU was observed in 24% of cases and 26% of controls with prior AU (*P* = .76). The most common category of inappropriate AU was inappropriate spectrum—accounting for 55% and 86% of inappropriate AU among cases and controls, respectively (Figure 1). No need for antibiotic therapy accounted for 27% of inappropriate AU among cases and 12% among controls. The most common indication for prescribed inappropriate antimicrobials was pneumonia (41% cases and 53% controls). There were no significant differences in mortality or LOS.

**Table 1.** Characteristics, antibiotic utilization and appropriateness, and outcomes, stratified by group

Baseline characteristic	Cases *N* = 102	Control *N* = 194	*P*-values
Age, years, median (IQR)	61 (52–71)	63 (54–71)	0.41
**Race/Ethnicity**			0.66
Non-Hispanic Asian	0 (0)	5 (3)	
Non-Hispanic Black	33 (32)	63 (32)	
Hispanic	45 (44)	80 (41)	
Other	8 (8)	12 (6)	
Unknown	4 (4)	7 (4)	
Non-Hispanic White	12 (12)	27 (14)	
**Sex**			0.81
Female	50 (49)	98 (51)	
Male	52 (51)	96 (49)	
**Location**			
Medical/surgical ward	76(75)	146 (75)	
Oncology	12(12)	20 (10)	
Critical care	14(14)	28 (14)	
PPI use	62 (61)	118 (61)	0.99
Prior GI surgery	30 (29)	69 (36)	0.29
Prior *C. difficile* infection	7 (7)	14 (7)	0.91
**Any antibiotics, 30 days prior to index test**	92 (90)	168 (87)	0.37
Antibiotics recommended by ID	50 (54)	76 (45)	0.16
**Abx use was Inappropriate**	22 (24)	43 (26)	0.76
**Indications selected among inappropriate Abx use**			
Pneumonia	9 (41)	23 (53)	
Urinary tract infection	2 (9)	5 (12)	
Sepsis	2 (9)	4 (9)	
Skin soft tissue infection	2 (9)	4 (9)	
Intra-abdominal infection	1 (5)	3 (7)	
Surgical prophylaxis	3 (14)	0 (0)	
Bacteremia	2 (9)	1 (2)	
Unknown	1 (5)	2 (5)	
Osteomyelitis	0 (0)	1 (2)	
**Inappropriate antibiotics recommended by ID**	7 (32)	7 (16)	0.20
**Outcomes**			
30-day mortality	16 (16)	27 (14)	0.68
Length of stay, days, median (IQR)	29 (15–47)	26 (13–46)	0.94
Length of stay after index test, days, median (IQR)	12 (6–26)	9 (4–21)	0.43
**Antibiotic use (AU) metrics**			
Select antibiotics^*^ days of therapy, 30 days prior to index test	3 (0–7)	5 (1–9)	0.01
Select antibiotics^*^ AU rate (DOT per 1000 days present), within 30 days prior to index test	258 (0–500)	400 (67–750)	0.01
Days of therapy, all antibiotics 30 days prior to index test, median (IQR)	7 (3–13)	9 (4–15)	0.13
AU rate, all antibiotics 30 days prior to index test, DOT per 1000 days present	618 (200–1000)	800 (266–1167)	0.14

* Select antibiotics: cefepime, ceftriaxone, piperacillin-tazobactam, ciprofloxacin, and levofloxacin; all antibiotics: any antibiotic used within the specified time; DOT= days of therapy; AU= antibiotic use; PPI= protein pump inhibitor

## Discussion

This study found no association between inappropriate AU and HO-CDI. This result was unexpected given available data regarding CDI and AU. A 2014 meta-analysis found that exposure to antibiotics was associated with a 60% increased risk of CDI.^7^ A retrospective chart review of antibiotic courses prior to HO-CDI at two academic tertiary acute care hospitals in Ontario, Canada, found 45.5% of antibiotic courses were inappropriate.^4^ Another study of inpatient AU found that 56% of patients had AU that was not supported by clinical data.^8^

This study found inappropriate AU in 24% of cases and 26% of controls. This frequency of inappropriate AU is lower than other studies have reported. In studies by Srigley et al^4^ and Magill et al,^8^ inappropriateness categories were similar to those of our study. Clinical guidelines were used to identify AU that was not needed, was given longer than is recommended, or covered a wider spectrum of bacteria than is recommended. Those studies reviewed patients from 2011–2012 and 2015 and did not report frequency of ID consultation. Progress in antimicrobial stewardship practices since 2015,^9^ as well as frequent ID consultation in our population, might contribute to the finding of less inappropriate AU in our study.

Conflicting with prior studies, our control group had significantly higher rates of AU for five high-risk antibiotics compared to cases. However, the analysis of all antibiotics given prior to the index test showed no significant difference. Control selection might have contributed to this finding. *C. difficile* tests are typically ordered for patients with prior AU. Selection based on *C. difficile* orders may bias the control group toward a higher proportion of patients with antibiotic exposure compared to a random sample of admitted patients. Studies assessing AU and CDI should consider inclusion of a control group with no history of CDI test. Another reason for this unexpected result may be the lack of data on AU from outside institutions. Alternatively inappropriate AU might not be a key driver of HO-CDI in our institution. Infection control practices or patient colonization might also be important factors.^1^

Limitations of this study include its retrospective design, limited sample size, matching, and subjective judgment in determining appropriateness of AU. Matching can limit the potential for bias due to confounding but cannot eliminate it. The use of patient location in matching was intended to maintain similarities between the groups in broad categories such as oncology or critical care. With this strategy, we could not identify two controls for every single case. Matching on more specific patient characteristics might have increased similarity between the groups but would have made it more difficult to match controls to cases. Although identification of “appropriate AU” was based on clinical judgment, questionable cases were adjudicated by additional investigators. One strength of our study was detailed review of patient charts to determine appropriateness of AU in addition to reporting overall AU rate. Another strength is the applicability of our study to hospitals that serve a population with a substantial proportion of Black and Hispanic patients.^10^

In conclusion, this study did not find more inappropriate or total AU in HO-CDI cases compared to CDI negative controls. This finding may reflect advances in antimicrobial stewardship practices, frequent ID consultation in our institution, or the choice of control group in the study. Nevertheless, opportunities remain for overall reduction in inappropriate AU.

## Supporting information

10.1017/ash.2025.10161.sm001 John et al. supplementary material John et al. supplementary material

## Supplementary material

The supplementary material for this article can be found at https://doi.org/10.1017/ash.2025.10161.
