# Supplementary material for: C. Diff-erently: evaluating the association of inappropriate antibiotic use with hospital-onset Clostridioides difficile infection
Source: Antimicrob Steward Healthc Epidemiol. 2025 Oct 8;5(1):e255. doi: 10.1017/ash.2025.10161 (PMC12538348; doi:10.1017/ash.2025.10161)
Supplement: John et al. supplementary material [file S2732494X25101617sup001.pdf]

Supplementary Appendix

A. Definition of Inappropriate Antibiotic Use

Antibiotic not needed: Antibiotic therapy not required due to non-infectious cause of symptoms/syndrome (e.g. shortness of breath due to fluid overload secondary to decompensated heart failure)

Prolonged antibiotic duration: Administered duration of therapy longer than recommended for the suspected or confirmed infectious syndrome

Inappropriate antibiotic spectrum: Use of antibiotics covering a different spectrum of bacteria than is recommended for the suspected or known syndrome, or using redundant antibiotics. For empiric therapy, a prior culture with a resistant organism or with Pseudomonas would support appropriateness of using an empiric antibiotic covering that organism. For therapy after culture results were available, use of broader antibiotic spectrum than was needed for the bacteria identified in cultures from the suspected source of infection would be considered inappropriate.

Montefiore

Montefiore Antimicrobial Stewardship Program (ASP)

Syndrome Specific Guidelines
(Antibiotic initiation, adult inpatients)

Notes:
- ***Guideline is not intended to replace clinical judgment***
- ***For most syndromes, this guideline offers initial dose recommendations only, ongoing dose and frequency may depend on renal function and weight*** (e.g., IV vancomycin, gentamicin, acyclovir, SMX/TMP, etc.)
- Always send 8-10cc/ blood cx bottle as part of the initial fever workup
- Look at prior micro results to help guide you
- Recommendations may be amended during drug shortages
- Syndromes are listed in alphabetical order
- ***ID assistance is recommended for severely ill patients, compromised hosts, pregnancy, etc.***

Abbreviations:
- MDRO = multidrug resistant organism
- PCN = penicillin
- Abx = antibiotics
- UCx = urine cultures; BCx = blood cultures

Take a "time out" at 72hrs after starting antibiotics
- ✓ Is this antibiotic still needed?
- ✓ Can it be narrowed in spectrum or switched to PO?
- ✓ How long do I plan to treat?
- ✓ Have I obtained appropriate diagnostics and followed up on results?
- ✓ Did I document antibiotic plan in the EMR?
- ✓ At transfer to another unit or discharge, did I communicate the correct REMAINING duration of antibiotics to avoid excess use?

Clarifying an Antibiotic Allergy

- ✓ **Non-IgE mediated penicillin reaction:** non-urticarial rash, injection site reaction, unknown/remote reaction (e.g., type IV, delayed hypersensitivity reaction)
- ✓ **IgE mediated/immediate hypersensitivity reaction:** (requires prior drug exposure) urticarial rash, dyspnea, hoarseness, bronchospasm, facial/tongue swelling, anaphylaxis
- ✓ 1 in 10 patients report an PCN allergy but 8 in 10 are no longer allergic after a 10-year period
- ✓ PCN-cephalosporin cross-reactivity rate: ≤ 2.5%; benefit of cephalosporin likely outweighs risk
- ✓ *Take opportunity to challenge while in monitored setting* - ***look back at administered meds from prior admits to see if β-lactam ever given → if no reaction, you are good to go!***

Colonization vs. True Infection

Colonization may predispose to infection, but does NOT always indicate active infection, and treatment does not prevent future infection:

- ✓ ***Asymptomatic* pyuria and bacteriuria are common in elderly females and nursing home residents (altered mental status and falls are NOT symptoms of UTI)**
- ✓ Is the patient symptomatic with signs of active infection? (ex. dysuria, purulent sputum, fever, leukocytosis)
- ✓ Are symptoms persistent > 24 hours?
- ✓ Is this a condition that may not require abx or only a short course of abx? (ex: tracheitis, aspiration pneumonitis)
- ✓ Do radiographs support the presence of infection?
- ✓ Was the catheter changed on schedule?
- ✓ Is there a single dominant organism in culture with many WBC and low epithelial cells?
- ✓ Are antibiotics alone likely to cure the infection? Has source control been achieved?
- ✓ Can always call ID/ASP for assistance

Aspiration

Obtain CXR, CBC, sputum culture if antibiotics required (aspiration is often caused by chemical irritation, not infectious process; treatment may not be required)

Refer to Montefiore Respiratory Infection Guidelines

Catheter-associated Bloodstream Infection

Send at least 2 sets of blood cultures (culprit line and peripheral blood), remove the line, and send tip for culture

Treatment

- IV Vancomycin 15-20mg/kg + Cefepime 1-2g
- **If severe PCN allergy:** IV Vancomycin 15-20mg/kg +/- Aztreonam 1-2g
- **If endocarditis is suspected remove the line, consult ID, and order TEE*
- *ID consult recommended for Staphylococcus aureus, Candida spp., Pseudomonas spp., and MDROs*

Clostridioides difficile Infection (CDI)

Obtain CBC, BMP, abdominal Xray or CT if ileus, stool *C. difficile,* **STOP** unnecessary PPI, antibiotics, laxatives; *Surgery/GI/ID consult recommended for severe or fulminant disease*

Refer to Montefiore *C. difficile* Guidelines

COPD Exacerbation

Refer to Montefiore Respiratory Infection Guidelines

Community-Acquired Pneumonia

Refer to Montefiore Respiratory Infection Guidelines

Hospital-Acquired Pneumonia

Refer to Montefiore Respiratory Infection Guidelines

Influenza

Obtain Influenza/RSV PCR, SARS-CoV-2 PCR to distinguish between viral syndromes, CXR; place in “droplet isolation”

Treatment (for patients at risk for severe illness and symptom onset within 72h): Oseltamivir (CrCl ≥ 60 ml/min: 75mg PO Q12h, CrCl 30-59 ml/min: 30mg PO Q12h, CrCl ≤ 29ml/min: 30 mg PO Q24h, HD 30mg after HD)

Severe influenza with respiratory failure in an ICU patient: consider ID consult for IV peramivir

COVID-19

Obtain SARS-CoV-2 PCR, CXR, admission labs, and CT thorax as indicated; place patient in “special pathogens precautions” isolation (N95, gown gloves, eye protection)

Refer to NIH COVID-19 Treatment Guidelines

Intra-abdominal Infection (non-CDI)

Community acquired: Ceftriaxone IV 1-2g (2g for BMI>30) + Metronidazole 500mg IV/PO, OR Cefoxitin 1-2g IV/PO +/- Metronidazole 500mg IV/PO, OR Ciprofloxacin 400mg IV/500mg PO + Metronidazole 500mg IV/PO (severe PCN allergy)

- ✓ Note: q12h dosing of Metronidazole is appropriate for most indications (except amebiasis and C. *difficile* infection)

Severe Sepsis/Septic Shock or Risk for MDROs (extended hospital stay, extensive outpatient antibiotic exposure): Piperacillin/tazobactam 4.5g IV (Aztreonam IV 1-2g + Metronidazole 500mg IV/PO if severe PCN allergy + Vancomycin 15-20mg/kg IV for Streptococcal/Enterococcal coverage)

Meningitis/Encephalitis

Obtain LP, blood cultures, CT/MRI; **ID consult recommended**

Meningitis:

- Age <50 **AND** normal host immunity: Vancomycin 15-20mg/kg IV Q8-Q24h + Ceftriaxone 2g IV Q12h
- Age >50 **OR** Immunosuppressed: Vancomycin 15-20mg/kg IV Q8-Q24h + Ceftriaxone 2g IV Q12h + Ampicillin 2g IV Q4h (if normal kidney function; dose adjust for diminished GFR, page ID/ASP for assistance)

Suspect HSV Encephalitis:

Acyclovir 10 mg/kg IBW (or adjusted body weight for BMI >30) every 8 hours (if normal renal function; page ID/ASP for assistance); add to meningitis regimen above in at-risk patient if coverage of both meningitis and encephalitis required

Anaphylaxis to Penicillin:

Vancomycin 15-20mg/kg x IV + [Levofloxacin 750mg IV or Ciprofloxacin 400mg IV Q8-12h]

- If *Listeria* coverage is needed, add SMX-TMP 5mg/kg q12h

Neutropenic Fever

Look for focal sx/signs on exam and history, blood cultures, UA/UCx, CXR, CT especially if prolonged neutropenia

- ✓ Look back at clinical cultures from prior admits to select a targeted antibiotic regimen

Treatment: Cefepime 2g IV

*If patient is hemodynamically unstable, or concern for multidrug resistant infection, begin meropenem 500 mg and consult ID

MMC Criteria for adding IV Vancomycin

- Evidence of pneumonia on imaging
- Skin or soft tissue infection
- Suspected central line infection
- Known recent prior MRSA infection
- Gram positive bacteremia
- Septic shock

Severe Penicillin allergy

Aztreonam 2g IV +/- tobramycin 5-7mg/kg IV q24h (if c/f MDRO or severe sepsis) + Vancomycin 15-20mg/kg IV per nomogram, **for intra-abdominal source, can add metronidazole 500mg IV*

Staphylococcus aureus bacteremia:

Remove catheter if present, obtain daily blood cultures, TTE and potentially TEE

ID consult strongly recommended for assistance with work up, investigation for distant sites of infection, and management (sometimes dual antibiotic therapy, ophthalmology consult, additional tests like PET scan are recommended); OPAT follow-up recommended on discharge

Skin & Skin Structure/Bone Joint Infections

- ✓ *Obtain nares MRSA PCR which has negative predictive value >90% for MRSA clinical infection (e.g., if negative, can discontinue IV vancomycin)*

- ✓ ***If patient is clinically stable and infection is chronic; hold antibiotics to increase bone/tissue culture yield***

- ✓ Here is a 5 – year retrospective review of microorganisms isolated from wound/abscess/tissue cultures from MMC's micro lab (may be biased to sicker patients who had cultures sent); **note low *Pseudomonas aeruginosa* prevalence <15%**

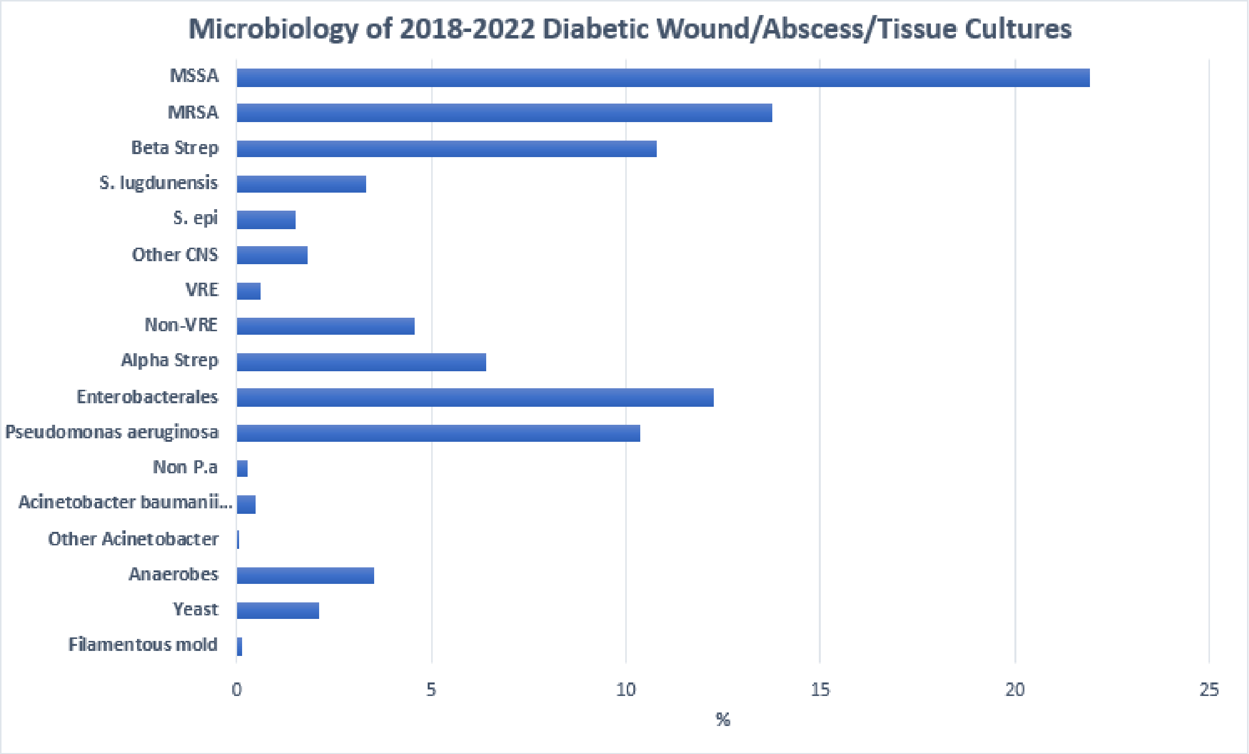

Syndrome	Regimen
**Non-purulent cellulitis**<br><br>*Non-purulent, streaky/diffuse = Streptococcus species*	**Mild:**<br>PO: Cephalexin 500 mg *OR* cefuroxime 500 mg *OR* amoxicillin 500 mg<br><br>**Moderate:**<br>IV: Cefazolin 1-2 g *OR* penicillin G potassium 4 million units
**Purulent cellulitis**<br><br>*Purulent cellulitis = Staphylococcus aureus*<br><br>*Note high local prevalence of MRSA	**MRSA:**<br>Mild:<br>PO (Preferred): TMP/SMX 1-2 DS tabs *OR* doxycycline 100 mg<br>PO (Alternative): clindamycin 600 mg<br><br>Moderate:<br>IV: Vancomycin 1 g<br><br>**MSSA:**<br>Mild:<br>PO: Cephalexin 500 mg *OR* cefuroxime 500 mg *OR* amoxicillin/clavulanate 875 mg *OR* dicloxacillin 500 mg<br><br>Moderate:<br>IV: Cefazolin 1-2 g
**Severe SSTI/ suspected necrotizing fasciitis**	Vancomycin 15-20 mg/kg IV + piperacillin/tazobactam 4.5 g IV<br><br>If suspected necrotizing fasciitis:<br>**Call Surgery/ID consult**, add **clindamycin 900 mg IV** to severe SSTI regimen above (refine later based on cultures); **during clindamycin shortage, IV linezolid 600 mg** can be used – *will cover MRSA AND neutralize GAS toxin*
**Diabetic Foot Infection**	Chronic wound with no evidence of cellulitis or systemic signs of infection: **hold antibiotics**<br><br>Mild:<br>PO: Amoxicillin/clavulanate 875 mg *OR* cefdinir + metronidazole *OR [if anaphylaxis to penicillin]* ciprofloxacin + clindamycin<br><br>Moderate:<br>*No recent hospitalizations or IV antibiotics, no history of Pseudomonas aeruginosa or MDR organisms*<br>Ceftriaxone + metronidazole *OR* Ampicillin-sulbactam 3 g IV<br><br>Severe or complicated DFI, history of *Pseudomonas aeruginosa* or MDR organisms<br>Piperacillin/tazobactam 4.5 g *OR* cefepime + metronidazole<br><br>History of MRSA/positive MRSA nares PCR

	Add vancomycin 1 g
**Osteomyelitis**	Obtain CRP, ESR with routine labs, X-ray (or MRI if inconclusive), tissue/bone cultures if possible (superficial wound cultures may not be accurate)<br><br>**Chronic osteomyelitis without evidence of cellulitis or systemic signs of infection: Hold antibiotics to increase bone/tissue culture yield to guide therapy**<br><br>Mild to Moderate:<br>• Ceftriaxone 2g (+/- metronidazole 500mg if necrotic, foul smelling or C. acnes suspected for upper extremity infections) **OR** Ampicillin/sulbactam 3g<br>• +/- Vancomycin 15-20mg/kg (if prior MRSA, +MRSA PCR, or excess past abx exposure)<br><br>Sepsis OR Suspect *P. aeruginosa* (i.e., foot puncture wound, water exposure, excess abx, past Pseudomonas):<br>• Piperacillin/tazobactam 4.5g IV **OR** Cefepime 1-2g IV (+/- metronidazole 500mg if necrotic, foul smelling)<br>• +/- Vancomycin 15-20mg/kg IV (if high MRSA risk)<br><br>Severe Penicillin allergy:<br>• Aztreonam 1-2g IV **OR** ciprofloxacin 400mg IV **OR** levofloxacin 750mg IV<br>• +/- Vancomycin 15-20mg/kg IV (GP coverage)<br>• +/- metronidazole 500mg IV if necrotic, foul smelling

Urinary Tract Infection

Change foley, obtain UA/UCx, U/S of kidneys if suspect pyelonephritis or obstruction, BCx if febrile or meets sepsis criteria

Cystitis: Cephalexin 500mg PO, **OR** TMP/SMX 1 DS tab PO, **OR** Nitrofurantoin 100mg PO (for CrCl >30ml/min), **OR** Cefdinir 300mg PO, **OR** gentamicin 3mg/kg IV IBW x 1 (Amikacin 10 mg/kg IV IBW x 1 If suspected or confirmed *Pseudomonas aeruginosa*), **OR** Ciprofloxacin 500mg PO (severe PCN and sulfa allergy).

- For urine isolates, cefazolin results predict results for the oral agents like cefdinir, cefpodoxime, cefuroxime, and cephalexin when used for therapy of uncomplicated UTI due to *E. coli, K. pneumoniae, P. mirabilis*
- For outpatients or patient's being discharged, call patient's pharmacy to make sure prescribed antibiotic is in stock, *otherwise there will be a treatment delay*

Complicated UTI/pyelonephritis (without h/o MDRO): Ceftriaxone 1g IV

- **Anaphylaxis to Penicillin:** Gentamicin 3mg/kg IV IBW (Amikacin 15 mg/kg IV IBW if suspected or confirmed *Pseudomonas aeruginosa*), OR Aztreonam 1-2g IV, OR Ciprofloxacin 400mg IV or 500mg PO (if from home ONLY)

Suggested Antibiotic Durations

Syndrome	Median Duration
COPD exacerbation, meets criteria for antibiotics	3-5 days
CAP	5 days
Complicated CAP (empyema, bacteremia, S. aureus PNA, abscess, Legionella)	Duration variable up to several weeks (ID consult recommended);<br>7-21 days for Legionella based on severity
HAP/VAP (empiric treatment OR isolation of specific pathogen such as MRSA, Pseudomonas, MDRO, etc.)	7 days
Bacterial meningitis	7-21 days depending on organism isolated (ID consult recommended)
HSV encephalitis	14-21 days (ID consult recommended)
Catheter-related bloodstream infection (catheter removal recommended for source control)<br><br>For *Staph aureus*, Pseudomonas, Yeast, and/or recurrent bacteremia – ID consult recommended	**CoNS**: 5-7 days if transient; longer if persistent<br>***S. aureus***: up to 4-6 weeks<br>**GNB** (not Pseudomonas): 7-14 days if neg BCx and source controlled<br>***Candida spp.*:** at least 14 days from first neg BCx; 6 weeks or more for endocarditis
Influenza	Oseltamivir 5 days; up to 7-10 days only if critically ill
Uncomplicated UTI<br><br>Pyelonephritis/complex UTI	3-5 days<br><br>7-10 days; ≥14 days if renal abscess (ID consult rec.)
Intra-abdominal source	4-7 days **if source controlled**
Skin and soft tissue<br>(if discrete lesion drained, often no further abx needed)	Pathogen/case specific; 5 to ≥ 14 days<br>if systemic illness, deep infection, non-healing, unusual pathogen, compromised host – ID and Surgery input suggested
C. difficile colitis	10 days for first or second episode<br>See Montefiore *C. diff* guideline for details
Osteomyelitis	4-6 weeks depending on source control/hardware; ID consult and OPAT referral recommended
Neutropenic fever<br>(ID consult suggested)	Hold Abx once afebrile ≥ 48h with negative cultures, resolving neutropenia; if documented source, treat accordingly for site and organism

Shorter Is Better

Diagnosis	Short (d)	Long (d)	Result	#RCT
CAP	3-5	5-14	Equal	14
Atypical CAP	1	3	Equal	1
Possible PNA in ICU	3	14-21	Equal	1*
VAP	8	15	Equal	2
cUTI/Pyelonephritis	5 or 7	10 or 14	Equal	9**
Intra-abd Infection	4	10	Equal	2
Complex Appendicitis	2	5	Equal	1
GNB Bacteremia	7	14	Equal	3†
Cellulitis/Wound/Abscess	5-6	10	Equal	4‡
Osteomyelitis	42	84	Equal	2
Osteo Removed Implant	28	42	Equal	1
Debrided Diabetic Osteo	10-21	42-90	Equal	2φ
Septic Arthritis	14	28	Equal	1
AECB & Sinusitis	$\leq$5	$\geq$7	Equal	>25
Variceal Bleeding	3	7	Equal	1
Neutropenic Fever	AFx72h/3 d	+ANC>500/9 d	Equal	2
Post Op Prophylaxis	0-1	1-5	Equal	55Ψ
Erythema Migrans (Lyme)	7	14	Equal	1
P. vivax Malaria	7	14	Equal	1
**Total: 19 Conditions**				**>125 RCTs**

*Infiltrate on CXR but low CPIS score (≤6), both ventilated and non ventilated, likely CAP, HAP, and VAP combined; **2 RCT included males, the smaller one found lower 10-18 d f/up cure in males with 7 days of therapy but no difference at longer follow-up, larger exclusive male study found no diff in cure; †GNB bacteremia also in UTI/cIAI RCTs; ‡3 RCTs equal, 1 (low dose oral flucox) ↑relapses 2° endpoint; φall patients debrided, in 1 study total bone resection (clean margins); ΨIncludes meta-analysis of 52 RCTs; refs at https://www.bradspellberg.com/shorter-is-better

Source:

https://www.bradspellberg.com/shorter-is-better

Montefiore

Montefiore Respiratory Infection Guidelines

(Revised 2023, includes CAP, HAP/VAP, aspiration, lung abscess/empyema, COPD exacerbations; adapted from IDSA/ATS or NYS, CDC, NIH guidelines)

General notes:

- "De-label" penicillin allergy when possible and update medical record if patient able to tolerate beta-lactams
- Reassess patient condition daily and switch to PO whenever possible
- Do NOT "restart the antibiotic clock at discharge," 5 days for CAP = 5 days, not 5 MORE days after discharge

Highlights:

- No role for routine steroids (outside of severe cases requiring ICU admission)
- Obtain respiratory and blood cultures ONLY in severe disease and those who receive empiric MRSA or *Pseudomonas aeruginosa* coverage
- Procalcitonin is not recommended to determine need for initial therapy but can assist in monitoring response to treatment
- MRSA nasal swab is recommended to guide de-escalation when empiric MRSA coverage is started
- Routine follow up X-rays not recommended for bacterial PNA
- COVID-19 guidelines not included here, please visit https://www.covid19treatmentguidelines.nih.gov/therapeutic-management/

Risk factors for MRSA or *P. aeruginosa* (obtain nasal MRSA PCR; respiratory culture and nasal influenza/RSV swab as indicated; note that *S. aureus* PNA was observed in COVID-19 patients at MMC as a complication of prolonged intubation; if COVID-19 suspected, obtain SARS-CoV-2 PCR):

- Prior isolation of either on cultures
- Hospitalization AND treatment with IV antibiotics in prior 90 days
- IVDU (specifically MRSA)
- Pneumonia associated with influenza (specifically S. aureus and Streptococci)

Treatment

Outpatient CAP: non-severe, no risk factors for antibiotic resistant pathogens (e.g. MRSA, *P. aeruginosa*). Pathogens: *Streptococci, Haemophilus*, atypicals like *Mycoplasma, Legionella* (esp. warmer months)

- Amoxicillin 1g three times daily, OR
- Doxycycline 100mg twice daily
- Azithromycin 500mg on day 1, then 250mg daily for remaining course (ONLY recommended in areas with macrolide resistance <25%) – *Note MMC microbiology laboratory does not routinely test for S. pneumoniae susceptibilities vs. macrolides*

Inpatient CAP: non-severe, no risk factors for antibiotic resistant pathogens (e.g., MRSA, *P. aeruginosa*). Pathogens: see above.

- Combination beta-lactam (ceftriaxone 1-2g IV daily) with a macrolide (azithromycin 500mg PO daily) OR doxycycline 100mg PO twice daily (if contraindication to macrolide or quinolone); **OR**
- Monotherapy with respiratory fluoroquinolone (levofloxacin 750mg) *especially if anaphylaxis to beta-lactams*
    - 750mg dose recommended for those with normal renal function (CrCl >50mL/min) in order to maximize concentration-dependent killing properties of fluoroquinolones; elderly patients and those with diminished renal function may require lower dose
- Azithromycin IV and Levofloxacin IV/PO dose of 750mg require ID/stewardship approval *on initiation*

Inpatient severe CAP but no risk factors for MRSA or *P. aeruginosa*

- Combination beta-lactam (ceftriaxone 1-2g IV daily) with a macrolide (azithromycin 500mg IV daily); **OR**
- Combination beta-lactam plus a respiratory fluoroquinolone (levofloxacin 750mg PO daily if CrCl >50mL/min (or equivalent dose adjusted for renal function)); *lower quality of evidence than bullet 1*

Inpatient severe CAP with risk factors for MRSA (see above)

- Ceftriaxone 1-2g IV daily + Azithromycin 500mg IV daily + Vancomycin 15-20mg/kg IV (or linezolid 600mg IV every 12 hours, or ceftaroline 600mg IV every 12 hours)
- ID/stewardship approval is required for vancomycin IV beyond 72 hours and azithromycin IV. ID consult is required for PO/IV linezolid or ceftaroline *on initiation*

- Severe allergy to penicillin: Levofloxacin 750mg IV daily if CrCl >50mL/min (or equivalent dose adjusted for renal function) + Vancomycin 15-20mg/kg IV

Inpatient severe CAP/HAP/VAP with risk factors for *P. aeruginosa* (see above)

- Piperacillin/tazobactam 4.5g every 8 hours extended infusion over 4 hours if CrCl >20mL/min (or equivalent dose adjusted for renal function) **OR** Cefepime 2g every 8 hours if CrCl >60mL/min (or equivalent dose adjusted for renal function)
    - *If in ICU, extended infusion of piperacillin/tazobactam and cefepime is recommended*
- Severe allergy to penicillin: Levofloxacin 750mg IV daily if CrCl >50mL/min (or equivalent dose adjusted for renal function) + Vancomycin 15-20mg/kg IV +/- Tobramycin 7mg/kg IV ideal body weight (for added Pseudomonal coverage)
- *Contact ID/Stewardship if prior history of multidrug resistant Pseudomonas*

Inpatient severe CAP/HAP/VAP with septic shock, ARDS +/- ECMO, unknown organism; compromised host

- Add azithromycin 500mg IV to above regimen to cover atypicals (*Legionella, Mycoplasma* spp.)
- If urine *Legionella* Ag (for serogroup 1) is negative, azithromycin IV can be continued for other *Legionella* and *Mycoplasma* species at discretion of ID consult

Inpatient severe pneumonia w/ lung abscess or empyema

- ID consult strongly advised for assistance with work up and treatment; may contact antimicrobial stewardship for initial recommendations
- Pathogens can include *S. aureus, Streptococcal* species, *Haemophilus* species, Gram negatives, anaerobes, or Mycobacteria

Aspiration pneumonia (cover oral *Streptococcus* and other oral flora):

- Regimens: ampicillin/sulbactam 3g IV every 6 hours; amoxicillin/clavulanate 875mg/125mg PO twice daily if CrCl >30mL/min (or equivalent dose adjusted for renal function); clindamycin 300-600mg PO/IV every 6-8 hours

COPD Exacerbations

- **Suggested work-up:** chest X-ray, sputum culture if bacterial infection suspected, influenza/RSV PCR if in season, SARS-CoV-2 PCR
- **GOLD Criteria for antibiotics:**
    - Sputum purulence and either increased sputum volume or dyspnea **OR**

  - Severe disease requiring positive pressure ventilation
- **Mild (treated with short acting bronchodilators only):**
  - No antibiotics recommended
- **Moderate (treated with short acting bronchodilators plus antibiotics and/or corticosteroids):**
  - Doxycycline 100mg twice daily **OR** Azithromycin 500mg daily
- **Severe (requiring hospitalization or emergency room visit):**
  - **No risk factors for *P. aeruginosa:***
    - **PO:** Amoxicillin/clavulanate 875mg/125mg twice daily if CrCl >30mL/min (or equivalent dose adjusted for renal function), **OR** cefdinir 300mg twice daily, **OR** Levofloxacin 750mg daily (severe PCN allergy) if CrCl >50mL/min (or equivalent dose adjusted for renal function)
    - **IV**: Ampicillin/sulbactam 3g every 6 hours, **OR** Ceftriaxone 1-2g daily, **OR** Levofloxacin 750mg daily (severe PCN allergy) if CrCl >50mL/min (or equivalent dose adjusted for renal function)
  - **Risk factors for *P. aeruginosa***: chronic colonization or prior isolate of *P. aeruginosa* (particularly within the past 12 months), very severe COPD (FEV_1 <30% predicted), bronchiectasis on chest imaging, intravenous broad-spectrum antibiotic use within the past 3 months, chronic systemic glucocorticoid use
    - **PO:** Levofloxacin 750mg daily if CrCl >50mL/min (or equivalent dose adjusted for renal function)

      IV: Piperacillin/tazobactam 4.5g every 8 hours extended infusion over 4 hours if CrCl >20mL/min (or equivalent dose adjusted for renal function), **OR** Cefepime 2g every 8 hours if CrCl >60mL/min (or equivalent dose adjusted for renal function), **OR** Levofloxacin 750mg daily if CrCl >50mL/min (or equivalent dose adjusted for renal function) (severe PCN allergy)

Duration of Antibiotics

	**Duration**	**Comments**
**CAP (not *Legionella,* MRSA, or *Pseudomonas*)**	5 days	None
**CAP with *Legionella,* MRSA, or *Pseudomonas***	*Legionella* – 7-21 days<br>*MRSA and P. aeruginosa* – 7 days or more, may depend on host factors and other complications	ID consult advised for work up and treatment recommendation
**Aspiration pneumonitis**	3-5 days	Some cases may not require antibiotics (chemical pneumonitis)
**HAP/VAP**	7 days	Includes treatment of MDROs

**Empyema or lung abscess**	~3-4 weeks of treatment (IV or PO) and possible need for reimaging to confirm adequate improvement	ID consult advised for antibiotic selection/duration, esp. for XDR organisms; refer to OPAT upon discharge
**COPD exacerbation**	5-7 days	None

Oral Step-down Options

**Suspect *P. aeruginosa***	**Suspect MRSA**	**Ampicillin-sulbactam or ceftriaxone started**
• Levofloxacin 750mg PO daily if CrCl >50mL/min (or equivalent dose adjusted for renal function)	• Doxycycline 100mg PO twice daily<br>• Linezolid 600mg twice daily	• Amoxicillin/clavulanate 875mg/125mg PO twice daily if CrCl >30mL/min (or equivalent dose adjusted for renal function)<br>• Cefdinir 300mg PO twice daily

Levofloxacin Dosing Table

**Creatinine Clearance (mL/min)**	**Dose***
>50	750mg IV/PO daily
20-49	750mg IV/PO q48h
10-19	750mg IV/PO x 1, then 500mg q48h
HD	750mg IV/PO x 1, then 500mg after each HD
CVVH	750mg IV/PO q48h

*** 750mg dose requires ID/antimicrobial stewardship approval**
